# Antibiofilm property and multiple action of peptide PEW300 against *Pseudomonas aeruginosa*

**DOI:** 10.3389/fmicb.2022.963292

**Published:** 2022-07-29

**Authors:** Meng Wang, Zifeng Deng, Yanmei Li, Keyong Xu, Yi Ma, Shang-Tian Yang, Jufang Wang

**Affiliations:** ^1^School of Biology and Biological Engineering, South China University of Technology, Guangzhou, China; ^2^Kaiping Healthwise Health Food Co., Ltd, Kaiping, China; ^3^Guangdong Provincial Key Laboratory of Fermentation and Enzyme Engineering, South China University of Technology, Guangzhou, China; ^4^Department of Chemical and Biomolecular Engineering, The Ohio State University, Columbus, OH, United States

**Keywords:** antimicrobial peptides, *Pseudomonas aeruginosa*, antibiofilm, Cecropin A, mode of action

## Abstract

*Pseudomonas aeruginosa* (*P. aeruginosa*), an opportunistic pathogen, is often associated with difficulties in treating hospital-acquired infections. Biofilms formed by *P. aeruginosa* significantly improve its resistance to antimicrobial agents, thereby, posing a great challenge to the combat of *P. aeruginosa* infection. Antimicrobial peptides (AMPs) have recently emerged as promising antibiofilm agents and increasingly attracting the attention of scientists worldwide. However, current knowledge of their antibiofilm behavior is limited and their underlying mechanism remains unclear. In this study, a novel AMP, named PEW300, with three-point mutations (E9H, D17K, and T33A) from Cecropin A was used to investigate its antibiofilm property and antibiofilm pathway against *P. aeruginosa*. PEW300 displayed strong antibacterial and antibiofilm activity against *P. aeruginosa* with no significant hemolysis or cytotoxicity to mouse erythrocyte and human embryonic kidney 293 cells. Besides, the antibiofilm pathway results showed that PEW300 preferentially dispersed the mature biofilm, leading to the biofilm-encapsulated bacteria exposure and death. Meanwhile, we also found that the extracellular DNA was a critical target of PEW300 against the mature biofilm of *P. aeruginosa*. In addition, multiple actions of PEW300 including destroying the cell membrane integrity, inducing high levels of intracellular reactive oxygen species, and interacting with genomic DNA were adopted to exert its antibacterial activity. Moreover, PEW300 could dramatically reduce the virulence of *P. aeruginosa*. Taken together, PEW300 might be served as a promising antibiofilm candidate to combat *P. aeruginosa* biofilms.

## Introduction

Pathogens including *Pseudomonas aeruginosa* (*P. aeruginosa*), *Staphylococcus aureus*, *Enterococcus faecium*, *Acinetobacter baumannii*, *Klebsiella pneumonia,* and *Enterobacter* species (called “ESKAPE”) infections have already been a significant problem of hospital infections and seriously threaten public health owing to their inherent antibiotic resistance (Zhen et al., 2019b; De Oliveira et al., 2020). Biofilms are surface-associated microbial communities embedded in self-produced extracellular polymeric substances (EPSs; Lee and Zhang, 2015). Biofilm formation is beneficial for bacteria survival under adverse environmental conditions and is implicated in the majority of bacterial infections (Thi et al., 2020). With the powerful protection of biofilms, these pathogens can attach to inert surfaces of medical devices, living tissues, and implanted prostheses, and successfully “escape” the damage of antibacterial agents (Ciofu and Tolker-Nielsen, 2019; Tagliaferri et al., 2019; Thi et al., 2020). The severity of pathogen infections and the invalidation of antibiotics have resulted in high morbidity and mortality (Founou et al., 2017; Zhen et al., 2019a; Manandhar et al., 2020; Yungyuen et al., 2021).

*Pseudomonas aeruginosa*, a Gram-negative (G^−^) bacterium, is an opportunistic human pathogen associated with clinical and chronic infections such as urinary tract infections, chronic wound infections, ventilator-associated pneumonia, and biofilm-related systemic infections (Serra et al., 2015; Shortridge et al., 2019; Abbott et al., 2020; Garcia-Clemente et al., 2020). Epidemiological surveillance reports from the European Centre for Disease Prevention and Control (ECDC) and the International Nosocomial Infection Control Consortium (INIC) revealed that *P. aeruginosa* was the most prevalent bacteria isolated from clinical samples in ICU-acquired pneumonia episodes and ICU-acquired bloodstream infections (Pang et al., 2019). As a member of the “ESKAPE” pathogens, *P. aeruginosa* has already been stipulated as a “critical” pathogen among the bacterial pathogens list of the World Health Organization (WHO), emphasizing the urgent need for the exploration and development of novel antibacterial agents to combat *P. aeruginosa* biofilms (Tacconelli et al., 2018).

In recent decades, antimicrobial peptides (AMPs) have been regarded as promising therapeutics against biofilm-forming pathogens due to their broad-spectrum antibacterial activity and inability to induce resistance (Cardoso et al., 2019; Di Somma et al., 2020; Hancock et al., 2021). Several studies on bacteria in the planktonic state have shown that AMPs adopt a unique membrane-targeting mechanism of action, unlike conventional antibiotics with specific targets, which disturb bacterial membranes mainly by interaction with negatively charged phospholipids and cause cell death (Chen et al., 2020; Li et al., 2020a; Luna et al., 2021). Significantly different from bacteria in the planktonic state, the control of biofilm-encapsulated bacteria is very difficult because of their extremely low permeability to antimicrobial agents (Gilbert et al., 2002; Hall and Mah, 2017). Previous studies have shown that biofilms could increase antibiotic resistance by up to 1,000-fold compared with planktonic bacteria (Kouidhi et al., 2015). However, these studies have mostly focused on the development of AMPs on bacteria biofilms, study on antibiofilm mechanism is still in its infancy. Hence, thorough and systematic studies on the antibiofilm mechanisms of AMPs are urgent priorities to accelerate the clinical development of AMPs as antibiofilm agents.

In our previous research, a novel AMP named PEW300 was designed by three mutations (E9H, D17K, and T33A) from Cecropin A (an natural AMP that is possible to use in medical and agricultural fields as a new and safe biocontrol agent), which had shown strong antimicrobial activity against several Gram-positive (G^+^) and G-bacteria (Wang et al., 2019; Hashemi et al., 2021). In this study, PEW300 was utilized to explore the antimicrobial and antibiofilm effect on *P. aeruginosa* and reveal its mechanism of action. Initially, the physicochemical properties, hemolysis, and cytotoxicity of PEW300 were assessed. Then, we studied the antimicrobial and antibiofilm ability of PEW300 on *P. aeruginosa* and determined its antibiofilm pathway. To explore the potential targets of PEW300, the mature biofilm components, cell membrane integrity, intracellular disturbance effected by PEW300 were then investigated. Considering that virulence factors are the main culprits of bacterial infections, we also explored the impact of PEW300 on *P. aeruginosa* virulence production. This study may provide a new reference for the research on the antibiofilm mechanisms of AMPs and demonstrate that PEW300 have good potential to be a safe and efficient antibacterial agent to combat *P. aeruginosa* biofilms.

## Materials and methods

### Strains, chemicals, and peptide preparation

*Pseudomonas aeruginosa* JCM5962 was preserved in our laboratory; *E. coli* DH5α and BL21(DE3) competent cells were purchased from Tiangen Biotech (China). Dulbecco’s-modified eagle medium (DMEM) and Fetal bovine serum (FBS) were purchased from Gibco (United States). The crystal violet, kanamycin, and gentamicin were obtained from Sangon Biotech Co. (China). Fluorescein isothiocyanate-labeled concanavalin A (FITC-ConA), 4′,6-diamidino-2-phenylindole (DAPI), Nile red, and SYPRO red were purchased from Sigma-Aldrich Co. (USA). The N-Phenyl-1-naphthylamine (NPN) and 3,3′- Dipropylthiadicarbocyanine iodide (DiSC_3_-5) were purchased from Aladdin (China). All other chemicals and reagents used in this study were of reagent grade.

PEW300 peptide was produced by our previous established protein expression and purification system (Wang et al., 2018). In this system, a high yield of AMPs can be acquired by simple centrifugation, with no expensive steps like NTA affinity chromatography and high-performance liquid phase separation. Purified PEW300 was dialyzed to PBS buffer and stored at-80°C for further experiment.

### Antibacterial and antibiofilm assays

The minimum inhibitory concentration (MIC) was assessed by the previously described method (Wiegand et al., 2008). Antibiofilm assays contain inhibition of biofilm formation assay and dispersion of preformed biofilm assay. The biomass of biofilm was quantified by the crystal violet stain method (Qi et al., 2020). In inhibition of biofilm formation assay, bacteria mixed with different concentrations (0, 5, 10, 20, 50, and 100 μg/ml) of PEW300 were loaded into a sterile 96-well plate and incubated at 37°C for biofilm formation (without shaking). Twenty four hours later, the supernatant was discarded and residual planktonic cells were washed with PBS twice. The biomass of biofilm was quantified by the crystal violet stain method. In dispersion of preformed biofilm assay, the preformed biofilms were incubated with different concentrations of PEW300 peptide (0, 5, 10, 20, 50, 100 μg/ml) at 37°C and the crystal violet stain method was performed to quantify the remaining biofilm. For cell viability detection, the mature biofilms were scraped with the pipette and sonicated for 10 min under low power condition (60 W) to release the viable bacteria. The sonicated suspensions were diluted and 50 μl was coated on a Mueller-Hinton agar plate. After incubation at 37°C for 16 ~ 18 h, the CFUs were counted.

### Hemolytic test, cytotoxicity, and drug resistance assays

For hemolytic test, the erythrocytes separated from mice blood were washed twice with 0.9% saline solution and then treated with 100 μl serial dilutions of PEW300 for 1 h. The hemolysis activity was determined by the hemoglobin content obtained from the absorbance of the supernatant at 570 nm after centrifugation. PBS and 0.1% Triton X-100 treatment were served as negative (0 hemolysis) and positive (100% hemolysis) controls. Human embryonic kidney 293 (HEK293) cells were adjusted to 1 × 10^5^ cells/ml with DMEM and dispensed into 96-well plates per 100 μl. Subsequently, increased concentrations of PEW300 (8 ~ 276 μg/ml) were added into 96-well plates separately and incubated with 5% CO_2_ for 48 h at 37°C. Cell viability was examined by cell counting kit-8 (CCK-8) assay (Beyotime, China). The drug resistance of *P. aeruginosa* to PEW300 and gentamicin were evaluated by the sequential passaging method according to the previous description (Huang et al., 2020). Briefly, *P. aeruginosa* cells were cultured to log phase and diluted to 2 × 10^5^ CFU/ml. Then, sub-MIC concentrations (1/2 MIC) of PEW300 and gentamicin, respectively, were incubated with the above bacterial suspension for 24 h at 37°C (*P. aeruginosa* grows for approximately 12 generations). These steps were repeated until 300 generations of growth were obtained. The number of generations was calculated from the value of log_2_ (bacterial concentration/2 × 10^5^). After every 24 h, the MIC values of PEW300 and gentamicin against *P. aeruginosa* were determined as described above.

### Circular dichroism measurement

The Circular dichroism (CD) spectra of PEW300 were measured on a Chirascan qCD Spectrometer (Applied Photophysics, United Kingdom) with wavelengths ranging from 190 to 260 nm using a 1 mm path length cuvette in 30 mM SDS and double-distilled water, respectively. Spectra were recorded with a band-width of 1 nm, a duration time of 1 s, and a scan speed of 100 nm/min. Each measurement was repeated three times to calculate the mean value. The spectra from the solvent were subtracted as background in data analysis.

### EPS analysis

After biofilm formation in 96-well flat-bottomed plate, the wells were washed with PBS twice and 100 μl of PEW300 at a concentration of 60 μg/ml were added into the wells and incubation for 24 h at 37°C. After incubation, the treated biofilms were washed with PBS and subsequently stained with 100 μl of the following fluorescent dyes (1:500 diluent of SYPRO red for proteins, 50 μg/ml FITC-ConA for carbohydrates, 0.5 μg/ml DAPI for extracellular DNA (eDNA), and 20 μM Nile red for lipids) at room temperature in the dark. The stained biofilms were visualized under a Leica DMI 6000 microscope (Germany).

Mature biofilms treated with increased concentrations (0, 10, 20, 40, 60 μg/ml) of PEW300 were incubated at 37°C for 24 h. Subsequently, EPS was extracted using a previous reported sonication method (Li et al., 2020c), and the amounts of carbohydrates, eDNA, and proteins were then analyzed. Quantitation of carbohydrates was applied by the phenol-sulfuric acid method as previously reported (Kim and Park, 2013). The amount of eDNA was determined using a Quant-iTTM PicoGreen R dsDNA Assay Kit (Invitrogen, United Kingdom). The protein content in EPS was quantified by the Lowry method using a Stable Lowry Protein Assay Kit (Sangon Biotech, China).

### Lipopolysaccharides binding assay, outer membrane permeability assay, and inner membrane depolarization test

The Lipopolysaccharides (LPS) (from *E. coli* O111:B4, Sigma-Aldrich, United States) binding affinity of PEW300 was assessed by monitoring the bacteria growth (OD_600_) inhibition with PEW300 pretreated with different concentrations (0, 5, 10, 20, 40, 80, and 160 μg/ml) of LPS; Permeabilization of *P. aeruginosa* outer membrane (OM) was evaluated by detecting the changes in fluorescence emission intensity of NPN with increased concentrations (0, 20, 40, and 80 μg/ml) of PEW300 according to the previous reported method (Wang et al., 2020); The IM depolarizing ability of PEW300 (8.25, 17.5, 35, and 70 μg/ml) was detected by monitoring the changes in fluorescence emission intensity of the DiSC_3_-5 dye at excitation and emission wavelengths of 622 and 670 nm, respectively (Li et al., 2020b).

### Transmission electron microscopy and scanning electron microscopy analyses

For transmission electron microscopy (TEM) observation, log-phase *P. aeruginosa* were treated with 20 μg/ml PEW300 and incubated at 37°C for 0, 10, 30, and 120 min. Then, a drop of bacteria suspension was placed on the prepared carbon film copper mesh for 5 min, cells were negatively stained with 3% phosphotungstic acid (Aladdin, China) for 3 min and blotted dry. Unbound phosphotungstic acid was washed with deionized water twice. Then cells were viewed on a Talos L120C TEM (Thermo Fisher Scientific, United States). For scanning electron microscopy (SEM) analysis, 1 ml of the diluted *P. aeruginosa* suspension (OD_600_ ≈ 0.1) in cation-adjusted Mueller-Hinton broth (CAMHB) was cultured in a 24-well plate with round glass bottom at 37°C for 24 h. Later, the supernatant was discarded and the plate was washed by PBS three times, then the preformed biofilms were incubated with 50 μg/ml PEW300 at 37°C for 2, 4, 6, 8, and 10 h. Untreated biofilm served as the control. After incubation, biofilms were fixed in 2.5% glutaraldehyde (Aladdin, China) at 4°C overnight and dehydrated with a graded series (70%, 85%, 95%) of ethanol for 10 min and soaked in 100% ethanol for 20 min. Dehydrated biofilms were dried with liquid CO_2_ at the critical point using an Autosamdri-815 (Tousimis, United States). Eventually the biofilms were coated by a gold sputter coater and examined using a HitachiS-500 SEM (Hitachi, Japan).

### Flow cytometric assay

*Pseudomonas aeruginosa* cultured in Mueller-Hinton broth (MHB) were harvested and washed with PBS three times, followed by resuspended in PBS. 10 μg/ml of Propidium Iodide (PI) was added to the suspension and incubated at room temperature for 10 min. Afterward, 20 μg/ml of PEW300 peptides were added to the suspensions and incubated at 37°C for 10, 20, 30, 60, and 120 min, separately. After incubation, the unbound PI was removed by centrifugation at 5,000×*g* for 5 min and the samples were analyzed using a CytoFLEX flow cytometer (Beckman Coulter, United States).

### DNA binding assay and intracellular reactive oxygen species assay

About 200 ng of purified *P. aeruginosa* genome DNA was mixed with different amounts (0, 4, 8, and 16, 32 μg/ml) of PEW300 and incubated at room temperature for 10 min. The mixtures were electrophoresed in 1% agarose gels containing 0.5 μg/ml ethidium bromide and the DNA bands were visualized using a gel documentation and image analysis system (BLT, China). The intracellular reactive oxygen species (ROS) level in *P. aeruginosa* was performed as described previously (Xiao et al., 2020). Briefly, *P. aeruginosa* cells at log-phase were mixed with 40 mM of 2,7-Dichlorodihydrofluorescein diacetate (DCF-DA) and incubated at 37°C for 30 min. After incubation, the cells were washed with PBS twice and an aliquot of 90 μl of diluted cells (1 × 10^8^ CFU/ml) was mixed with 10 μl of different concentrations (5, 10, 20, and 40 μg/ml) of PEW300 in a 96-well plate. PBS and 1% Triton X-100 were used as negative and positive controls. Then, the DCF fluorescence intensity was recorded on a SpectraMax M2 plate reader using an excitation wavelength of 488 nm and an emission wavelength of 530 nm.

### Real-time quantitative PCR and evaluation of *Pseudomonas aeruginosa* virulence

*Pseudomonas aeruginosa* cells were diluted to 10^8^ CFU/ml with MHB, then incubated with 10 μg/ml of PEW300 at 37°C for 8 h, cells with no PEW300 treatment as control. Total RNA was extracted from 5 ml cultures. The RNA extraction was assessed using RNAprep Pure Cell/Bacteria Kit (TIANGEN, China) according to the manufacturer’s instructions. Real-time quantitative PCR (qPCR) was carried out using LightCycler^®^96 (Roche, Switzerland) with SYBR Premix Ex Taq II (Tli RNaseH Plus) according to the manufacturer’s instructions. The expression level of 16S rRNA was used to normalize that of other genes. All experiments were repeated at least three times, and the primer sequences used in this experiment are publicly available (Supplementary Table S1).

A549 cells were adjusted to 1 × 10^4^ cells/mL and 100 μl of cells were seeded into a 96-well plate and incubated at 37°C with 5% CO_2_ for 48 h. Cells containing different concentrations (0, 5, 10, and 20 μg/ml) of PEW300 were infected with *P. aeruginosa* at a multiplicity of infection (MOI) of 10 for 6 h and the cell viability was assessed by CCK-8 assay. Besides, 4 μM calcein-AM (Santa Cruz, United States) was incubated with A549 cells at a density of 1 × 10^5^ cells/dish for 20 min in D-Hanks buffer and observed using a Leica DMI 6000 microscope. For comparative analysis of virulence production (pyocyanin, elastase, and alginate) with or without PEW300 treatment, *P. aeruginosa* cells were adjusted to an initial OD_600_ at 0.1 and incubated with increasing concentrations (0, 10, and 20 μg/ml) of PEW300 at 37°C for 8 h. Elastolytic activity was assessed using a 1% skimmed milk plate as previously reported (Cowell et al., 2003); Pyocyanin content was determined by chloroform-hydrochloric acid extraction method as previously described (Xu et al., 2016); The amount of alginate was quantified using the borate-carbazole method with sodium alginate (Sigma, United States) as a standard (Knutson and Jeanes, 1968).

### Statistical analysis

Each experiment was performed in triplicate and the data were analyzed by SPSS 16.0 software (SPAA Inc., Chicago, IL, United States). The data were presented as the means ± standard deviation and the statistical significance was defined as *p* < 0.05.

## Results and discussion

### Characterization of PEW300 peptide

**Figure 1:**
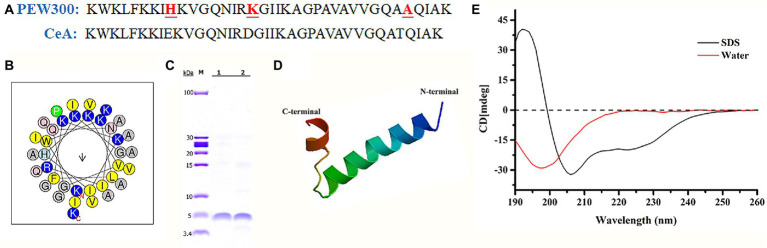
Physicochemical properties of the PEW300 peptide. **(A)** The sequences of PEW300 and Cecropin A (CeA). The red font and underline sites indicate mutation sites of the PEW300 peptide. **(B)** Predicted alpha-helical wheel of PEW300 peptide (https://heliquest.ipmc.cnrs.fr/cgi-bin/ComputParams.py). The hydrophobic residues are yellow, positively charged hydrophilic residues are blue, and non-charged polar residues are gray. **(C)** Purification of PEW300 peptide. Line 1 and line 2 are purified PEW300 peptides. **(D)** The secondary structure of PEW300 predicted by SWISS-MODEL (https://swissmodel.expasy.org/interactive). **(E)** CD spectra of the PEW300 peptide.

Compared with Cecropin A peptide, PEW300 owns three mutations in residues 9 (Glu to His), 17 (Asp to Lys), and 33 (Thr to Ala; Figure 1A). As predicted by Helical-wheel projection, PEW300 exhibited no negatively charged residues and was mainly composed of hydrophobic residues, non-polar residues, and positively charged residues, which is consistent with the general sequence properties of AMPs (Figure 1B). In addition, we analyzed the hydrophobicity and electrostatic potential of PEW300 surface, which showed its good amphipathic property and the strong positive charge at its N-terminus, indicating that PEW300 might possess higher antibacterial activity (Supplementary Figure S1). In this study, high purity of PEW300 peptide was acquired by our previously established peptide expression system (Figure 1C; Wang et al., 2018). The secondary structure of PEW300 as predicted by SWISS-MODEL showed it belongs to α-helical AMPs (Figure 1D). To investigate the structure of PEW300 in aqueous and mimic hydrophobic membrane environments, CD spectroscopy was performed. As shown in Figure 1E, PEW300 displayed a disordered structure in double-distilled water. In 30 mM sodium dodecyl sulfate (SDS), PEW300 exhibited two negative peaks at about 208 and 225 nm and a positive peak at about 192 nm, demonstrating a typical α-helical structure predisposition which is consistent with the predicted result (Figure 1D).

### Hemolytic activity, cytotoxicity, and drug resistance of PEW300

**Figure 2:**
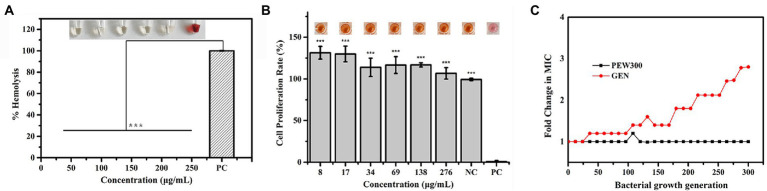
Hemolytic activity, cytotoxicity, and drug resistance of PEW300. **(A)** Hemolytic activity of PEW300 in mouse red blood cells. 0.1% Triton X-100 was used as the positive control (PC); ^***^p < 0.005 compared with PC group. **(B)** Cytotoxicity of PEW300 in HEK293 cells was determined by a CCK-8 assay. 0.1% Triton-X 100 and PBS were used as positive control (PC) and negative control (NC), respectively. Error bars represent the standard error from mean cell viabilities from three replicates, *** indicates *p* < 0.005 compared with PC group. **(C)** Drug resistance of the PEW300 and gentamicin. GEN, gentamicin.

As high hemolysis and cytotoxicity of AMPs are two critical factors that considerably hinder their further application (Zhu et al., 2020), we assessed the hemolytic activity and cytotoxicity of PEW300. As shown in Figure 2A, PEW300 showed negligible hemolytic activity at concentrations ranging from 50 to 250 μg/ml. The cell toxicity of PEW300 was determined using HEK293 cells by CCK-8 assay and the result showed that PEW300 had no cytotoxicity to HEK293 cells when ~276 μg/ml of PEW300 was used (Figure 2B). These results implied that PEW300 had good biosafety and might have good potential for further application. Although many studies had demonstrated that *P. aeruginosa* hardly developed drug resistance toward AMPs (Mahlapuu et al., 2016; de Breij et al., 2018), it is still necessary to assess the tendency of drug resistance of *P. aeruginosa* against PEW300. In the presence of sub-MIC levels of PEW300, we performed serial passage of nearly 300 generations of *P. aeruginosa* with no resistance as PEW300 continued to inhibit the growth of *P. aeruginosa* at MIC level (Figure 2C). However, the drug resistance of *P. aeruginosa* toward gentamicin appeared as early as the 36th generation and the MIC value increased approximately three times after 300 generations (Figure 2C). Taken together, these results indicated that PEW300 were less likely to cause *P. aeruginosa* resistance.

### Antibiofilm pathway of PEW300 against *Pseudomonas aeruginosa*

**Figure 3:**
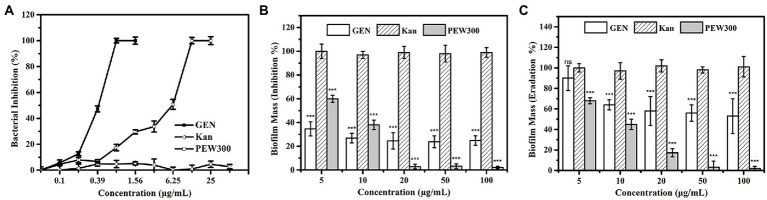
Antibacterial and antibiofilm properties of PEW300. **(A)** Antibacterial activity of PEW300, kanamycin (Kan), and gentamicin (GEN) against *Pseudomonas aeruginosa* JCM5962. **(B)** Inhibition and **(C)** dispersion activity of PEW300 against *P. aeruginosa* JCM5962 biofilms. Error bars represent standard error from mean values as determined by three repeated experiments, *** indicates *p* < 0.005 and ns means no significant difference compared with Kan-treated groups.

In our previous study, PEW300 showed a broad-spectrum antibacterial activity and inhibited most pathogenic bacteria including *Klebsiella pneumoniae*, *S. aureus*, *Staphylococcus epidermidis*, and *Bacillus cereus* among others with MICs between 4.93 to 28.35 μg/ml (Wang et al., 2019). Consistent with our expectations, PEW300 showed strong antimicrobial activity against *P. aeruginosa* with a MIC of 12.5 μg/ml (Figure 3A). Commonly used antibiotics in hospital such as gentamicin and kanamycin were selected as controls. As depicted in Figure 3A, compared with PEW300, gentamicin exhibited the strongest antimicrobial activity against *P. aeruginosa* (MIC value was 0.78 μg/ml), while kanamycin was ineffective with a MIC value greater than 100 μg/ml. In determination of antibiofilm ability, PEW300 showed a dose-dependent manner and approximately 98% of biofilm formation was inhibited when treated with 20 μg/ml of PEW300 (Figure 3B). Adopted the same manner, a dose-dependent dispersion activity of PEW300 on mature biofilm was observed and nearly 95% of mature biofilms were eradicated with 50 μg/ml of PEW300 treatment (Figure 3C). However, gentamicin was ineffective in both inhibition of biofilm formation and mature biofilm dispersion, especially as only 50% of mature biofilms were eradicated when treated with gentamicin at concentrations up to 100 μg/ml (Figures 3B, C). Previous studies demonstrated that the main cause of this failure of antibiotics against biofilms was the incomplete penetration of antibiotics into biofilm and thus caused the inability to interact with them (Roy et al., 2018; Sharma et al., 2019). Instead, AMPs could freely penetrate into biofilm because of their amphipathic properties and flexible structures, this might confer their antibiofilm ability. These results demonstrated that PEW300 exhibited excellent antibiofilm activity on *P. aeruginosa* biofilms.

**Figure 4:**
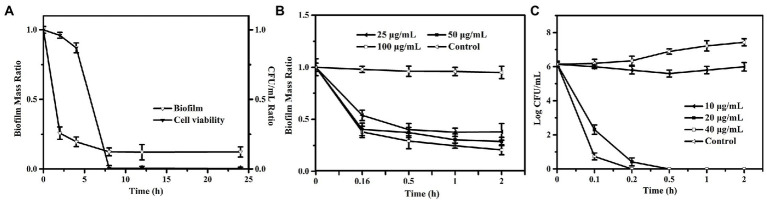
Antibiofilm pathway and time-killing kinetic analysis of PEW300 against *Pseudomonas aeruginosa*. **(A)** Antibiofilm pathway of PEW300 against *P. aeruginosa*. Reductive effect of PEW300 against *P. aeruginosa* biofilm **(B)** and planktonic cells **(C)**.

To investigate whether PEW300 act preferentially on the biofilm or the bacteria encased by biofilm, preformed biofilms were incubated with 50 μg/ml of PEW300 for 0, 2, 4, 8, 12, and 24 h. Cell viability and biofilm mass were analyzed at the same time. As depicted in Figure 4A, interestingly, we discovered PEW300 preferentially acted on the preformed biofilms, and nearly 60% of biofilms were eradicated within the first 2 h of incubation. Afterward, cell viability significantly decreased during 4 to 8 h of incubation and ~100% of cells were dead after 8 h of PEW300 treatment. To further understand the antibiofilm property of PEW300 on *P. aeruginosa*, a time-killing kinetic assay was also performed. As indicated in Figure 4B, PEW300 exhibited a dose-dependent dispersion of established biofilms and approximately 65% of the preformed biofilms were eradicated within 30 min, indicating that PEW300 has a rapid and efficient effect on the preformed biofilms. Similarly, PEW300 interacted with bacteria in a dose-dependent fashion and nearly 100% of bacteria were killed within 30 min (Figure 4C). Based on these findings, we speculate that PEW300 may preferentially act on the preformed biofilm and result in its degradation, the removal of biofilm led to the bacteria exposure that was then killed by PEW300 in a further incubation time.

**Figure 5:**
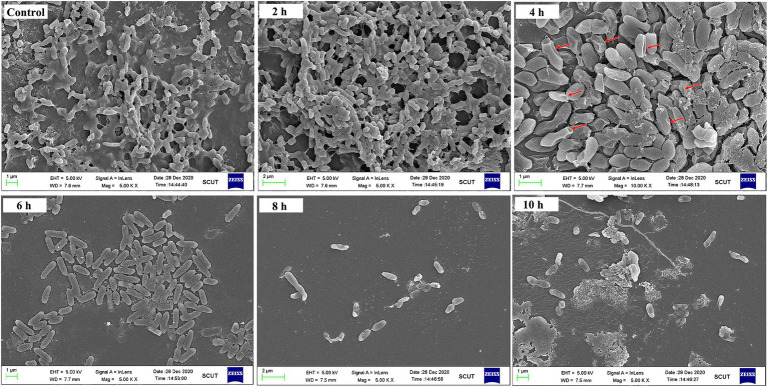
SEM observation of preformed biofilm of *Pseudomonas aeruginosa* JCM5962 affected by PEW300. The red arrow indicates the damaged JCM5962 cells.

To confirm this conclusion, an SEM observation was performed. As shown in Figure 5, in the absence of PEW300 treatment, massive biofilms enveloping *P. aeruginosa* cells were observed. After 2 ~ 4 h of PEW300 treatment, most biofilms disappeared and the encased *P. aeruginosa* cells were exposed. It is worth noting that the majority of bacteria were intact and no morphological abnormalities had been found within 2 h of PEW300 treatment. However, after 4 h of treatment, the cell morphology showed obvious wrinkles, fractures, and fragments, indicating that PEW300 destroyed the integrity of cell membrane. A significant reduction of *P. aeruginosa* cells was noticed with the prolongation of the incubation time (6 ~ 10 h; Figure 5). These results are consistent with the results of Figure 4A and confirm our speculations.

### Impact of PEW300 on the components of *Pseudomonas aeruginosa* biofilm

**Figure 6:**
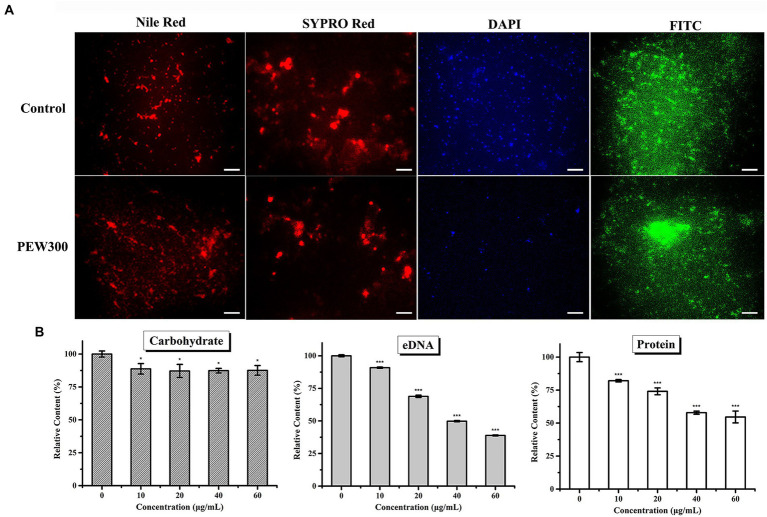
Eliminative action of PEW300 on *Pseudomonas aeruginosa* mature biofilm. **(A)** Fluorescence analysis of PEW300 on *P. aeruginosa* mature biofilm components. The scale bar is 200 μm. **(B)** Quantification analysis of mature biofilms components of *P. aeruginosa* JCM5962 affected by PEW300. * Indicates statistical significance compared to the control groups (^*^p < 0.05; ^***^p < 0.005).

Based on the above observations, we then investigated how PEW300 eradicated the preformed biofilms of *P. aeruginosa*. Four specific fluorescent dyes (FITC-ConA, Nile red, DAPI, and SYPRO red, which are separately able to bind to carbohydrates, lipids, eDNA, and proteins in biofilm) were utilized in this experiment. As shown in Figure 6A, PEW300 had a negligible impact on lipids and carbohydrates but showed degradation activity on eDNA and proteins, especially on eDNA component. To further confirm this observation, we also carried out quantitative analyses of the carbohydrate, eDNA, and protein components with or without PEW300 treatment. Consistent with fluorescence observation, the degradation activity of PEW300 on eDNA and protein components in mature biofilm was in a dose-dependent manner, ~75% and 45% of eDNA and proteins were separately eradicated when treated with 60 μg/ml of PEW300 (Figure 6B). Previous studies reported the AMPs could interact with eDNA and also could cleave eDNA *via* a nuclease-like activity (Zhang et al., 2020; Portelinha and Angeles-Boza, 2021). We speculate that PEW300 probably mainly degraded the eDNA *via* its amphipathic structure and cationicity and caused the collapse of the 3D architecture of biofilm, which eventually led to the significant destruction of the matured biofilm.

### Membrane disruption and the intracellular disturbance induced by PEW300

**Figure 7:**
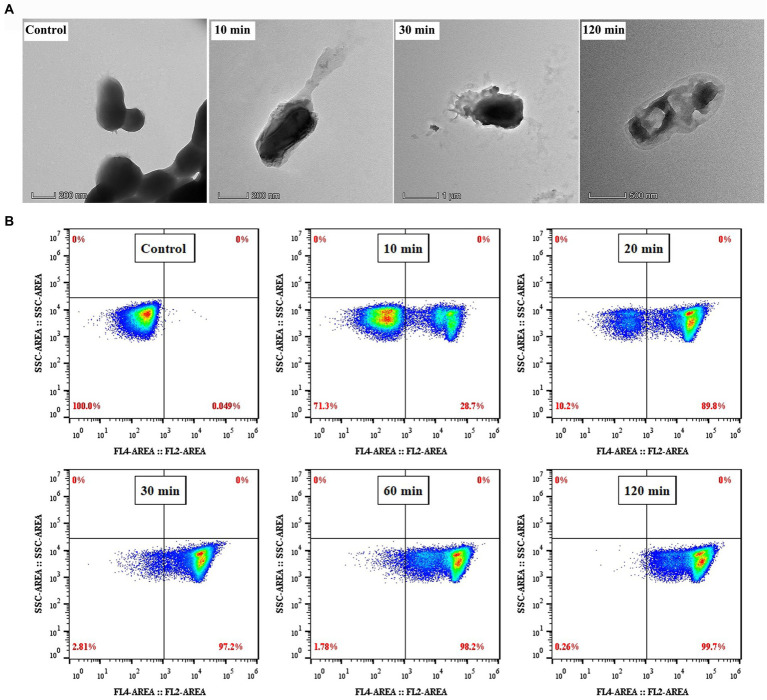
Disruption effect of PEW300 on *Pseudomonas aeruginosa* membrane. **(A)** TEM micrographs of *P. aeruginosa* JCM5962 treated with or without PEW300 (Control). **(B)** Flow cytometry analysis of the disruption ability of PEW300 on *P. aeruginosa* membrane.

Previous studies have reported that most AMPs could induce cell membrane disruption (Domingues et al., 2020; Liu et al., 2020; Wang et al., 2020). To explore whether PEW300 adopt the same manner, TEM observation was applied to directly observe the effect of PEW300 on the ultrastructure of *P. aeruginosa*. As shown in Figure 7A, the surface morphology of cells treated with PEW300 exhibited shrink, perforation, and mesosome-like structures while untreated cells were intact and smooth, indicating that PEW300 could destroy the cell membrane integrity, resulting in the extravasation of cytoplasmic content and enlargement of the extracellular matrix. To confirm the disruption effect of PEW300 on a membrane, a nuclear fluorescent probe PI which can traverse impaired cell membrane, combined with flow cytometry was applied. As shown in Figure 7B, untreated cells had almost no PI fluorescence signal (0.049%), indicating the intact bacterial membranes. While, the signals of cells treated with PEW300 (20 μg/ml) for 10 min was 28.7%, 20 min was 89.8%, and 30 min was 97.2%, indicating that the percentage of membrane rupture was in a time-dependent manner. As anticipated, nearly all *P. aeruginosa* cell membranes (99.7%) were ruptured when incubated with PEW300 for 2 h (consistent with the result of Figure 4C). These results demonstrated that PEW300 could destroy the integrity of *P. aeruginosa* cell membrane.

**Figure 8:**
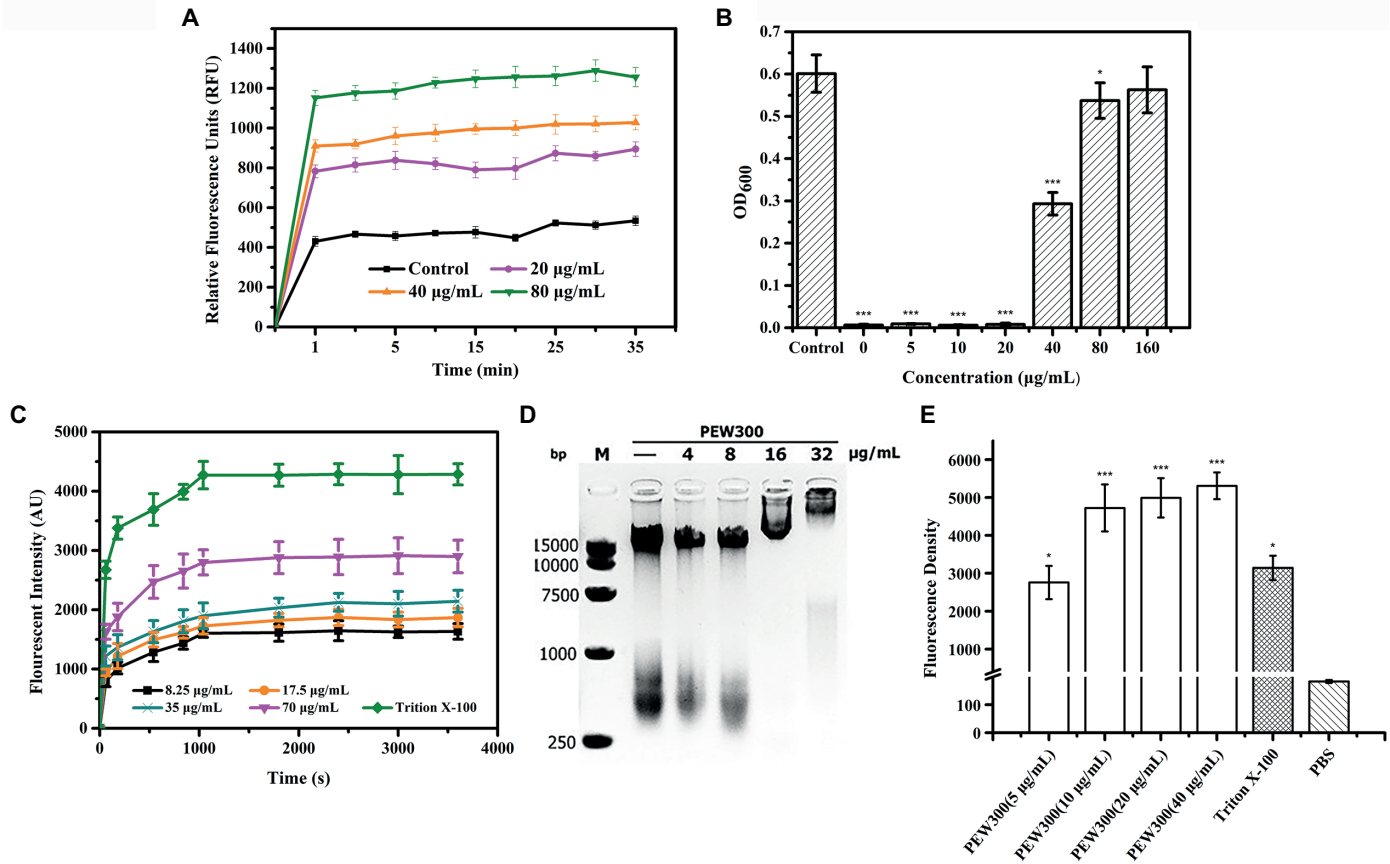
Membrane analysis and intracellular disturbance induced by PEW300. **(A)** Effects of PEW300 on OM permeabilization of *Pseudomonas aeruginosa* JCM5962. Control represents cells with no PEW300 treatment. **(B)** LPS-binding affinities of PEW300. Control means the growth of *P. aeruginosa* JCM5962 with no PEW300 and LPS incubation. ^*^p < 0.05, ^***^p < 0.005 compared with control. **(C)** The IM depolarization induced by PEW300. **(D)** The electrophoretic mobility shift analysis of the interaction between *P. aeruginosa* JCM5962 genomic DNA (200 ng) and PEW300 peptide. **(E)** Intracellular ROS intensity produced by *P. aeruginosa* JCM5962 in the presence of PBS, 1% Triton X-100, and series concentrations of PEW300 peptide. ^*^p < 0.05, ^***^p < 0.005 compared with PBS-treated group.

As we all know, the OM of G-bacteria is a robust permeability barrier to harsh environments (Domingues et al., 2020). β-lactam and quinolone antibiotics have no antibacterial activity against *P. aeruginosa* due to they cannot pass through the OM layer to reach their intracellular targets (Bruchmann et al., 2013; Torres et al., 2019; Hirsch et al., 2020). Thus, to study the permeabilization effect of PEW300, an environment-sensitive hydrophobic fluorescent probe NPN, which emits strong fluorescence in a hydrophobic environment and weak fluorescence in an aqueous environment, was used (Soh et al., 2020). As depicted in Figure 8A, the relative fluorescence units (RFU) of cells treated with PEW300 exhibited a sharp increase and reached a maximum within 1 min, showing that the OM permeabilization of *P. aeruginosa* caused by PEW300 was fast, and in dose-and time-dependent manner. LPS are the main components of the OM of G-bacteria (Chou et al., 2019) and we further explored the interaction between PEW300 and LPS. As shown in Figure 8B, the antibacterial activity of PEW300 was unaffected when treated with 0 ~ 20 μg/ml of LPS. However, when treated with 40 μg/ml of LPS, nearly 50% of the antimicrobial activity was lost, and the antimicrobial activity of PEW300 was completely disabled with 160 μg/ml of LPS treatment. The result suggested that PEW300 combined with LPS to exert the OM permeabilization. Unlike OM, the IM served as a barrier to protect the interior environment and played a key role in the transportation of nutrients and metabolites (Xie et al., 2018). Depolarization of IM will cause the release of DiSC_3_-5 and result in enhanced fluorescence (Zhu et al., 2020). As shown in Figure 8C, the IM depolarization of *P. aeruginosa* cells treated with PEW300 appeared in a dose-and time-dependent manner. The interaction between the negatively charged LPS and PEW300 led to an increase in OM permeability, which accelerated the permeation of PEW300 from the OM layer to the IM layer. Disruption of the IM induced by PEW300 led to the imbalance of electrical potential inside and outside the IM and eventually depolarization of the IM (Shao et al., 2018). These results confirmed that PEW300 could permeabilize the OM, depolarize the IM, and destroy the integrality of the cell membrane and then cause cell death.

Besides, we also tested the impact of PEW300 on *P. aeruginosa* genomic DNA. According to our assumption, if PEW300 is combined with genomic DNA, the migration of genomic DNA would be hindered in the agarose gel. As expected, the migration of genomic DNA appeared to be concentration-dependent and completely stayed in the gel pores when treated with 32 μg/ml of PEW300 (Figure 8D). These results suggested that PEW300 could not only act on the *P. aeruginosa* membrane but also on genomic DNA. Moreover, the intracellular ROS levels were also studied. As depicted in Figure 8E, there was a substantial increase in intracellular ROS in *P. aeruginosa* after incubation with different concentrations of PEW300 when compared with negative control (PBS treated cells), implying that PEW300 could cause the intracellular disturbance and result in the elevated ROS level. Consistent with the previous study (Xiao et al., 2020), this fairly high level of ROS within *P. aeruginosa* might be due to the disruption of the cell membrane (permeabilization of OM and depolarization of IM) or the interaction with genomic DNA by PEW300. All these results suggested that PEW300 exerted antimicrobial activity might through a multiple-action mechanism.

### PEW300 reduced the production of virulence factors of *Pseudomonas aeruginosa*

**Figure 9:**
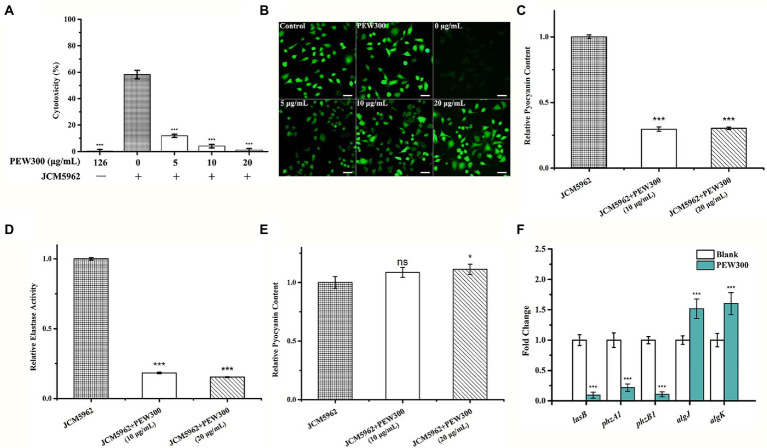
Analysis of *Pseudomonas aeruginosa* virulence factors affected by PEW300. PEW300 results in decreased virulence of *P. aeruginosa* JCM5962 in A549 cells characterized by **(A)** CCK-8 assay and **(B)** calcein-AM staining. ^***^p < 0.005 compared with *P. aeruginosa* JCM5962-treated group. Scale bar is 100 μm. Comparative analysis of **(C)** pyocyanin content, **(D)** elastase activity and **(E)** alginates content in *P. aeruginosa* JCM5962 treated with or without PEW300 peptide. **(F)** qPCR analysis of virulence-related gene expression. Data represent the mean ± SD from three independent experiments performed in triplicate. * indicates *p* < 0.05, *** indicates *p* < 0.005; ns means no significant difference compared with control.

As the virulence factors play a key role during the infection process of *P. aeruginosa*, we also investigated whether PEW300 could reduce the virulence of *P. aeruginosa*. Initially, a cytotoxicity experiment was performed. A549 cells were infected with *P. aeruginosa* mixture (containing increased concentrations of PEW300), and the cell viability was assessed by CCK-8 assay and calcein-AM staining. As shown in Figures 9A,B, the co-incubation of A549 with PEW300 (up to 126 μg/ml) had no impact on cell proliferation and viability while the co-incubation A549 with *P. aeruginosa* had resulted in nearly 60% of cells death. In addition, compared with the blank control group, the cytotoxicity of *P. aeruginosa* incubated with 5 and 10 μg/ml of PEW300 was dramatically decreased (5% ~ 10%) and was in a dose-dependent manner. Notably, the cell viability of *P. aeruginosa*-infected cells was high with almost complete survival of the cells (97.77%) when treated with 20 μg/ml of PEW300, implying that PEW300 could decrease the cytotoxicity of *P. aeruginosa*.

A previous study showed that elastase, pyocyanin, pyoverdine, and alginate are key virulence factors of *P. aeruginosa* for promoting its host pathogenicity (Xu et al., 2016). To further explore the effect of PEW300 on these virulence factors, we quantified the production of these virulence factors in *P. aeruginosa* cells with or without PEW300 treatment. As shown in Figures 9C–E, cells treated with 10 and 20 μg/ml of PEW300 both significantly decreased elastase and pyocyanin production, while the production of alginate was slightly increased. These results suggested that the reduced production of virulence factors caused by PEW300 might result in the decreased cytotoxicity of *P. aeruginosa*. In addition, we also evaluated the transcription levels of genes involving virulence factors expression by qPCR. As shown in Figure 9F, the elastase LasB encoding gene (*lasB*) and phenazine (intermediate metabolic product of pyocyanin) synthesis-related genes (*phzA1* and *phzB1*) were significantly downregulated in *P. aeruginosa* treated with PEW300; Besides, the expression levels of genes encoding alginate (*algK* and *algJ*) were upregulated in the PEW300-treated group. These results are consistent with virulence factors quantitation, demonstrating that PEW300 reduced the production of virulence factors of *P. aeruginosa* through downregulating the virulence-related gene expression.

As an important immunodominant molecule, LPS is involved in host cell attachment at the onset of infection and is essential for the virulence of many bacteria such as *P. aeruginosa* and *E. coli* (Jann and Jann, 1987; Pier, 2007), bacteria lacking LPS can diminish their virulence (Gupta et al., 1997; Davis and Goldberg, 2012). In addition, previous studies have reported that host innate immunity produces ROS during bacterial infection (Ramond et al., 2021). ROS include superoxide anions (O_2_^●-^), hydrogen peroxide (H_2_O_2_), and hydroxyl radicals (OH^●^), which damage bacterial cellular components, including DNA, membrane lipids and proteins, leading to cell death (Ezraty et al., 2017; Ramond et al., 2021). In the present study, the high intracellular ROS levels induced by PEW300 and the strong binding activity of PEW300 with *P. aeruginosa* LPS suggested two additional ways in which PEW300 reduces the virulence of *P. aeruginosa*.

**Figure 10:**
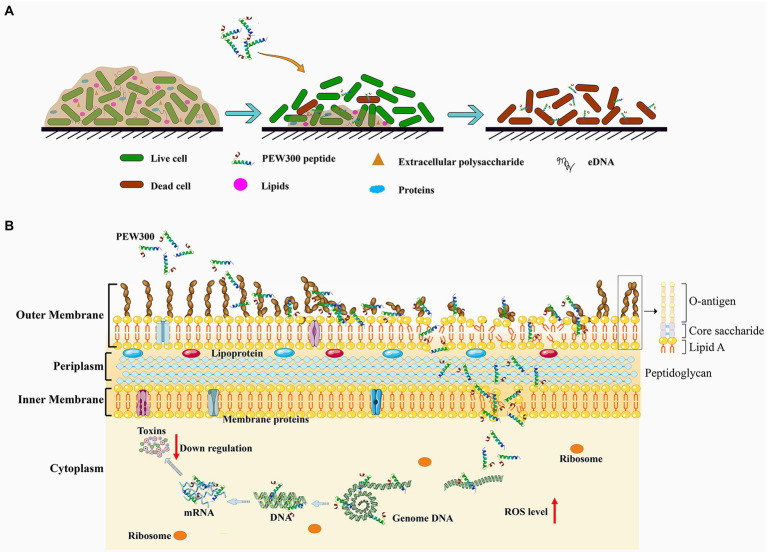
Schematic illustration of the **(A)** antibiofilm pathway and **(B)** the multiple actions of PEW300 against *Pseudomonas aeruginosa*.

To sum all, PEW300 adopted a unique mode of action to exert its antibiofilm activity, in which PEW300 preferentially eliminated the matured biofilm mainly by degradation of eDNA component and led to the wrapped bacteria exposure; then, the interactions between PEW300 and *P. aeruginosa* eventually resulted in the cell death (Figure 10A). In the process of interactions with *P. aeruginosa*, multiple actions of PEW300 were adopted to cause cell death, like increased the OM permeability, interacted with LPS, destroyed the integrity of cell membrane, and depolarization of IM; interacted with genome DNA, and caused high level of intracellular ROS. Besides, PEW300 also reduced the production of virulence factors (elastase, pyocyanin, pyoverdine, and alginate) by downregulating the virulence-related gene expression (Figure 10B).

## Conclusion

In the present study, we show that PEW300 has strong antibiofilm activity against *P. aeruginosa*. The excellent performance of PEW300 toward *P. aeruginosa* confers it as a potential alternative antimicrobial agent. Besides, our findings establish the antibiofilm mechanism of PEW300 against *P. aeruginosa* and provide a reference for the study of antibiofilm mechanism of other AMPs.

## Data availability statement

The datasets presented in this study can be found in online repositories. The names of the repository/repositories and accession number(s) can be found in the article/ Supplementary material.

## Author contributions

JW conceived the project and designed the experiment. MW performed the experimental work and wrote the first draft of the manuscript. ZD and YL performed the hemolytic assay and the cytotoxicity assay. KX analyzed the data. YM and S-TY reviewed and edited the manuscript. All authors contributed to the article and approved the submitted version.

## Funding

This work was supported by the Guangdong Major Project of Basic and Applied Basic Research (2020B0301030005).

## Conflict of interest

KX was employed by Kaiping Healthwise Health Food Co., Ltd.

The remaining authors declare that the research was conducted in the absence of any commercial or financial relationships that could be construed as a potential conflict of interest.

## Publisher’s note

All claims expressed in this article are solely those of the authors and do not necessarily represent those of their affiliated organizations, or those of the publisher, the editors and the reviewers. Any product that may be evaluated in this article, or claim that may be made by its manufacturer, is not guaranteed or endorsed by the publisher.
