# Supplementary material for: Antibiofilm property and multiple action of peptide PEW300 against Pseudomonas aeruginosa
Source: Front Microbiol. 2022 Jul 29;13:963292. doi: 10.3389/fmicb.2022.963292 (PMC9372277; doi:10.3389/fmicb.2022.963292)
Supplement: Supplementary file 1 [file Data_Sheet_1.docx]

Supplementary Material


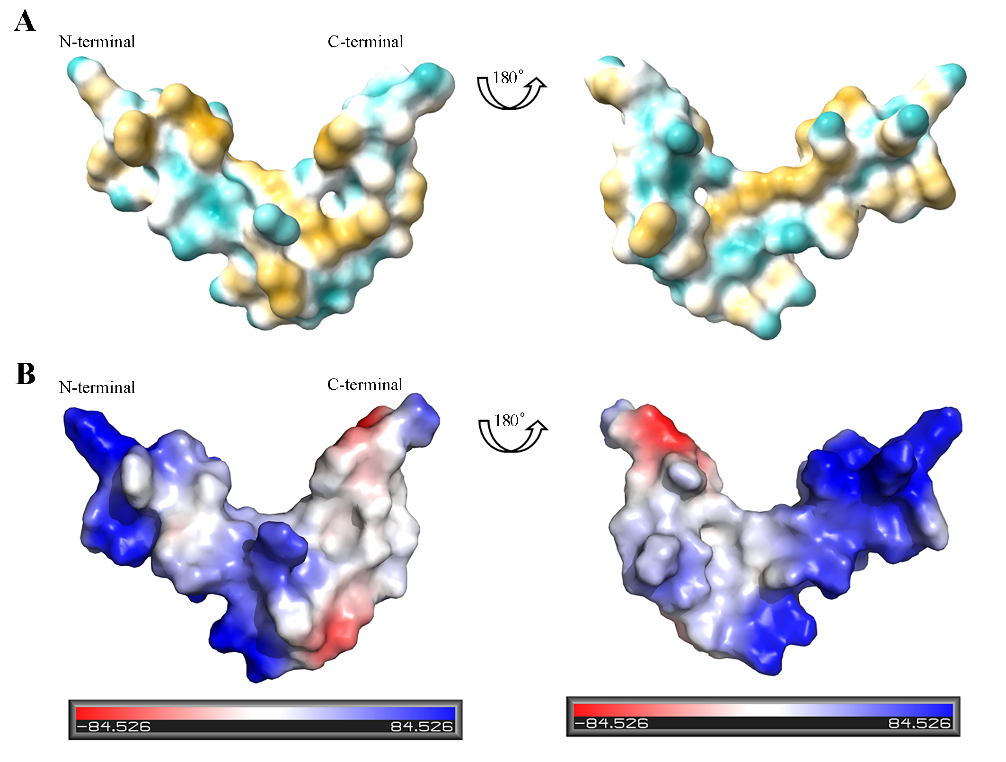


**Supplementary Figure S1.** The hydrophobicity and electrostatic potential of peptide surface were generated for PEW300. Peptide structures are represented as surface maps with a structural view of the front and back (180^o^ rotation). **(A)** The hydrophobicity of PEW300 surface was predicted by chimeraX software. Blue represents hydrophilic surface, yellow represents hydrophobic surface. **(B)** The vacuum electrostatics surface of PEW300 was generated by PyMOL software. Red represents negatively charged surface, gray represents neutral charged surface, and blue represents positively charged surface.

**Supplementary Table S1** Primers used in qPCR experiment.

| **Primers Name** | **Sequence** |
| --- | --- |
| *lasB-RT-F* | 5’-ATGTTCTATCCGCTGGTGTCG-3’ |
| *lasB-RT-R* | 5’-GCTGCCCTTCTTGATGTCGT-3’ |
| *phzA1-RT-F* | 5’-GTTACAGCGGCACAGCC-3’ |
| *phzA1-RT-R* | 5’-CGCACTCGACCCAGAAGT-3’ |
| *phzB1-RT-F* | 5’-ACGGCTGTGGCGGTTTA-3’ |
| *phzB1-RT-R* | 5’-CCGTGACCGTCGCATT-3’ |
| *algJ-RT-F* | 5’-GATACGCCACTGCTCAACG-3’ |
| *algJ-RT-R* | 5’-GAACAGCCAGCCGTCCT-3’ |
| *algK-RT-F* | 5’-CTGCAATACCCGCAGTCC-3’ |
| *algK-RT-R* | 5’-GCCAGTTCGTACCAGCACA-3’ |
